# Adiposity Phenotypes and Subclinical Atherosclerosis in Adults from Sub–Saharan Africa: An H3Africa AWI–Gen Study

**DOI:** 10.5334/gh.863

**Published:** 2021-03-19

**Authors:** Engelbert A. Nonterah, Michiel L. Bots, Abraham Oduro, Godfred Agongo, Cassandra C. Soo, Lisa K. Micklesfield, Felistas Mashinya, Palwendé R. Boua, Shukri F. Mohamed, Alisha N. Wade, Catherine Kyobutungi, Halidou Tinto, Shane A. Norris, Stephen M. Tollman, Michèle Ramsay, Diederick E. Grobbee, Kerstin Klipstein–Grobusch, Nigel J. Crowther, on behalf of the AWI–Gen and the H3Africa Consortium

**Affiliations:** 1 Clinical Sciences Department, Navrongo Health Research Centre, Ghana Health Service, Navrongo, GH; 2 Julius Global Health, Julius Center for Health Sciences and Primary Care, University Medical Center Utrecht, Utrecht University, Utrecht, NL; 3 Sydney Brenner Institute for Molecular Bioscience, Faculty of Health Sciences, University of the Witwatersrand, Johannesburg, ZA; 4 SAMRC Developmental Pathways for Health Research Unit, Faculty of Health Sciences, University of the Witwatersrand, Johannesburg, ZA; 5 Dikgale Health Demographic Surveillance Site, Department of Pathology and Medical Sciences, School of Health Care Sciences, Faculty of health Sciences, University of Limpopo, Polokwane, ZA; 6 Institut de Research en Sciences de la Santé, Clinical Research Unit of Nanoro, Nanoro, BF; 7 African Population and Health Research Centre (APHRC), Nairobi, KE; 8 SAMRC Rural Public Health and Health Transitions Research Unit (Agincourt), School of Public Health, Faculty of Health Sciences, University of the Witwatersrand, Johannesburg, ZA; 9 Division of Epidemiology and Biostatistics, School of Public Health, Faculty of Health Sciences, University of the Witwatersrand, Johannesburg, ZA; 10 Department of Chemical Pathology, National Health Laboratory Service, Faculty of Health Sciences, University of the Witwatersrand, Johannesburg, ZA

**Keywords:** Carotid intima-media thickness, subclinical atherosclerosis, obesity, adiposity, cardiovascular disease, sub-Saharan Africa

## Abstract

**Background:**

Obesity and adipose tissue distribution contribute to an increased risk of cardiovascular disease (CVD) by promoting atherosclerosis. This association has been poorly studied in sub–Saharan Africa (SSA) despite the rising prevalence of cardiovascular disease.

**Objectives:**

We determined the association between various adiposity phenotypes and carotid intima–media thickness (CIMT), a proxy of subclinical atherosclerosis, in a large SSA population.

**Methods:**

A population–based cross–sectional study was performed from 2013–2016 in Burkina Faso, Ghana, Kenya and South Africa. Body mass index (BMI), waist (WC), hip circumferences (HC), visceral (VAT) and subcutaneous adipose tissue (SCAT) using B-mode ultrasound were measured. Ultrasonography of left and right far wall CIMT of the common carotid artery was used as an indicator of subclinical atherosclerosis. Individual participant data meta–analyses were used to determine the associations between adiposity phenotypes and CIMT in the pooled sample while adjusted multivariable linear regression analyses were used for site specific analyses.

**Results:**

Data were obtained from 9,010 adults (50.3% women and a mean age of 50± 6years). Men had higher levels of visceral fat than women while women had higher BMI, waist and hip circumference and subcutaneous fat than men at all sites except Burkina Faso. In the pooled analyses, BMI (β–value [95% CIs]: 19.5 [16.8, 22.3] μm) showed the strongest relationship with CIMT followed by VAT (5.86 [4.65, 7.07] μm), SCAT (5.00 [2.85, 7.15] μm), WC (1.27 [1.09, 1.44] μm) and HC (1.23 [1.04, 1.42] μm). Stronger associations were observed in men than in women.

**Conclusion:**

Obesity within SSA will likely result in higher levels of atherosclerosis and promote the occurrence of cardio- and cerebrovascular events, especially in males, unless addressed through primary prevention of obesity in both rural and urban communities across Africa. The inverse association of VAT with CIMT in Burkina Faso and Ghana requires further investigation.

**Highlights:**

- All adiposity phenotypes were positively associated with common carotid intima–media thickness (CIMT) in the entire cohort (pooled analyses).
- BMI had the strongest association with CIMT compared to other phenotypes.
- The magnitude of association between adiposity phenotypes and CIMT was higher in men than in women.
- Subcutaneous adipose tissue was inversely associated with CIMT only in women.
- An unexpected finding was the inverse association of visceral adipose tissue with CIMT in Burkina Faso and Ghana.

## Introduction

Obesity is a major epidemic that occurs not only in the western world. Recent data from sub-Saharan Africa (SSA) demonstrate an equally high burden [1,2], with south and north Africa having the highest prevalence levels [3]. Projections from the World Health Organization (WHO) suggest that in 2025, 75% of the world’s obese population will be in low– and middle–income countries such as those in SSA [4,5]. The recent Non-Communicable Disease (NCD) Risk Factor Collaboration study of 112 million adults further observed that the rising prevalence of obesity in rural communities contributes significantly to the global obesity epidemic [6]. This is a major health concern, because obesity promotes the development of diabetes mellitus, is related to unfavorable levels of established cardiovascular risk factors such as dyslipidemia and hypertension, promotes the development of atherosclerosis and contributes to an increase in risk of symptomatic cardiovascular events [7,8].

Several measures of adiposity are available to assess various overall and central body fat phenotypes. Thus, for general obesity, body mass index (BMI) is the most common measure while total body fat can be measured using dual-energy X-ray absorptiometry (DXA). Central obesity is often assessed using waist circumference (WC), with specific abdominal fat depots i.e., visceral (VAT) and subcutaneous (SCAT) adipose tissue assessed using imaging techniques such as magnetic resonance imaging (MRI), computerized tomography (CT) and B-mode ultrasonography [9,10]. Peripheral or lower body obesity, particularly fat tissue in the gluteofemoral region, is commonly measured using hip circumference (HC) whilst gluteofemoral fat can be specifically measured using the imaging methodologies described above. These adiposity phenotypes have been suggested to exert different effects on the development of CVD risk factors and atherosclerosis [11]. Body fat distribution has been shown to be different in black Africans compared to their white counterparts [12,13].

The relationship between obesity and atherosclerosis can be investigated by the use of carotid intima-media thickness (CIMT) of the common carotid artery (CCA). Extensive research has shown that common CIMT, assessed using B-mode ultrasonography, is a marker of the presence of atherosclerosis locally and elsewhere in the arterial system, and, as such, is an intermediate stage for the development of CVDs [14,15,16]. However, most of the research examining the relationship of adiposity with atherosclerosis, assessed using common CIMT has been conducted in Whites [17], Asians [18,19,20] and African–Americans [21] from high income countries, but little information is available for black African populations from SSA.

In the present study, we used data from the Africa-Wits-INDEPTH [International Network for the Demographic Evaluation of Populations and Their Health] Partnership for Genomic Studies (AWI-Gen) project [22] to determine the relationship of various adiposity phenotypes with CIMT in four SSA countries – Kenya, Ghana, Burkina Faso and South Africa.

## Methods

### Study design, setting and participants

The AWI–Gen study is embedded within the NIH–funded Human Heredity and Health in Africa (H3Africa) Consortium. AWI–Gen is a population–based longitudinal study conducted in six sites in four SSA countries with baseline data collection occurring between 2013 and 2016 as described previously [23,24].

The study was conducted at five Health and Socio–Demographic Surveillance Sites (HDSS) under the INDEPTH Network and the MRC/Wits Developmental Pathways to Health Research Unit (DPHRU) in Soweto, South Africa [25]. The HDSS sites in South Africa were the cohort from Bushbuck Ridge in Mpumalanga (referred to as Agincourt HDSS in this publication) [26] and Dikgale HDSS [27]. The other sites were in East Africa: African Population and Health Research Center HDSS, Nairobi, Kenya [28] and two rural sites in West Africa: Nanoro HDSS, Burkina Faso [29] and the Navrongo HDSS, Ghana [30]. These countries are located in three sub-regional African blocks and may be representative of the social, geographical and genetic diversity of SSA.

Study participants were adult women and men aged 40–60 years living in the various sites. Details of the sampling methods and recruitment strategies used by the various sites have been described in a previous publication [24].

## Ethical considerations

The AWI-Gen study received ethics approval from the Human Research Ethics Committee (HREC) of the University of the Witwatersrand, Johannesburg, South Africa (Ethics approval identification number: M121029, renewed in 2017 with number: M170880). Additional ethics approvals were obtained from the national and institutional ethics boards/committees of the University of Limpopo, Nanoro HDSS, Burkina Faso; Nairobi HDSS, Kenya and Navrongo HDSS, Ghana. Written informed consent was obtained from all participants prior to recruitment.

## Data availability

The datasets generated and/or analyzed during the current study will be made publicly available in the European Genome–phenome Archive under the set of projects related to the Human Heredity and Health in Africa (H3Africa) Consortium. Details concerning access to data and DNA can be found in the document titled H3Africa Data and Biospecimen Access Committee Guidelines, available in the consortium documents section of the H3Africa website (www.h3africa.com).

## Data collection

The data collected included socio–demographic determinants of health, behavioral risk factors, and metabolic risk factors of CVDs, and the variables relevant to this paper are described briefly below.

### Outcome variable: Common CIMT

The outcome variable was the mean CIMT thickness in micrometers (µm) of the far walls of both the left and right CCA. Images were taken with a linear-array 12L-RS transducer using GE Healthcare B-mode LOGIQ *e* ultrasound machine (GE, Healthcare, CT, USA). To measure the right CCA, the participant was asked to lie down in a supine position with a pillow underneath the neck for slight extension, head turned towards the left at a 45-degree angle and gel applied to the exposed neck area. Using the two sternocleidomastoid muscles as landmarks, the exposed area was scanned along the longitudinal plane until the CCA was found. The operator then identified a continuous one-centimeter segment (10mm) of the CCA far wall after which the image was frozen. The operator then placed a cursor between two points (10mm apart) on this identified segment of the far wall with the proximal starting point one centimeter from the bulb of the CCA. The inbuilt software in the ultrasound equipment then automatically detected the intima-lumen and the media-adventitia interfaced and calculated the minimum, maximum and mean common CIMT in mm to two decimal places. To measure the left carotid, the participant’s head was turned to the opposite side, and the process was repeated [24]. We measured one site or angle of the CCA instead of multiple carotid sites or angles, because it was easier to measure and was equally reliable at enabling its widespread use at all study sites and aligned to real life setting measurements [31,32,33]. To ensure reproducibility and reduce CIMT measurement variability, masked repeated measurements of the 15 volunteers were conducted by each trainee and the lead trainer. The coefficient of variation between and within trainees was calculated and maintained at <2%. Subsequently, the same settings and calibrations of the ultrasound equipment were used at each site for data collection.

### Exposure variables: Adiposity phenotypes

The different adiposity phenotypes included in this analysis were body mass index, hip circumference, waist circumference, visceral adipose tissue and subcutaneous adipose tissue.

***BMI:*** Standing height to the nearest 0.1mm and weight to the nearest 0.1kg of each participant were measured without shoes and in light clothes using a Harpenden stadiometer (Holtain, Crymych, Wales) fixed to the wall and a digital calibrated weighing scale (SECA, Hamburg, Germany) respectively. The BMI was subsequently calculated as weight in kg/height in m^2^.

***Waist circumference (WC) and hip circumference (HC):*** WC was measured using a stretch-resistant tape measure (SECA, Hamburg, Germany). Participants were asked to wear only light or tight-fitting clothing, with the outer clothing removed to enable the tape to be positioned correctly. The participant breathed normally and stood straight with arms slightly abducted when the tape was placed horizontally around the narrowest part of the torso, about halfway between the iliac crest and the lowest rib. Measurements of the WC were taken at the end of a normal expiration without the compression of the tape. The WC was recorded to the nearest 0.1cm. The HC was measured by placing the tape around the most protruding part of the buttocks, ensuring that the zero mark was to the participant’s side. The HC was measured to the nearest 0.1cm.

***Visceral (VAT) and subcutaneous (SCAT) adipose tissues:*** These were measured using a B–mode LOGIQ *e* ultrasound machine (GE, Healthcare, CT, USA) with a 2.5MHz 3C–RS curved array transducer. A depth of 15cm and 9cm were used for VAT (medial) and SCAT measurements, respectively. For both measurements, participants were in the supine position, gel applied to the lower abdomen and the probe positioned with minimal compression on the midline at a level midway between the lower costal margin and the iliac crest with appropriate adjustments in the gain settings. The xiphi-sternum and umbilicus were used as a guide for accurate positioning. For VAT imaging, the transducer was held horizontal and the spine visualised in the horizontal position with the vertebra in the centre of the image. The participant was then asked to breathe quietly, and the measurement was taken at the end of the exhalation. To calculate the amount of VAT, the paused ultrasound image was brought up onto the screen. The first cursor was placed anterior to the spine (on the fat pad if visible), and the second cursor on the thin peritoneal layer beneath the anterior rectus abdominal muscles. Care was taken to ensure that the measurement was perpendicular to the surface of the lumbar vertebra and taken between the peritoneum and the spine where there is a clear space between the vertebra and the aorta. The measurement was repeated by producing a second image, and the results recorded in cm to two decimal places. An immediate quality check was done to ensure that the spine, abdominal aorta and rectus abdominal muscle were visualized on the image [34,35].

For the measurement of SCAT (transverse), the ultrasound probe was rotated through 90 degrees and the depth setting adjusted to 9cm. The rectus abdominus muscles were visualized, with care taken to ensure that both muscles were symmetrical in the image, and that the linea alba was centrally located, the gain adjusted accordingly, and the image captured. To calculate the SCAT measurement, the distance between the skin and the outer edge of the linea alba was measured on the screen, as described above. The measurement was then taken from a second image, and the results recorded similarly, in cm to two decimal places. The reliability of these ultrasound measurements in estimating adiposity has previously been validated in a black South African population [35].

### Covariates

Selected covariates were those that contribute to the development of CVDs and can confound the association between adiposity phenotypes and CIMT. These included age, level of education (highest level of education obtained), household socio-economic status (estimated by calculations based on household assets, according to the practice implemented by the Demographic and Health Surveys (DHS) Program) [24], alcohol consumption, smoking and physical inactivity assessed by measuring moderate-to-vigorous physical activity (MVPA). Physical inactivity was defined as MVPA < 150 mins/week. Other covariates included systolic blood pressure, glucose, total cholesterol, HDL-C, LDL-C and HIV infection and ART use. Post–menopausal status in women was defined as having no period within the past 12 months. Details of how these variables were measured have been reported previously [24].

## Data analysis

All data analyses were conducted with STATA version 14.1 (College Station, Texas, USA). We computed an average of the mean right and left far wall common CIMT thickness in micrometers (µm) as the main outcome variable. Continuous data were normally distributed and were summarized as means with standard deviations (±SD) and categorical data presented as absolute counts with corresponding percentages (%). Differences in mean (±SD) levels of the various adiposity phenotypes between women and men within the sites were determined using Student t-tests.

We determined the association between each adiposity phenotype and CIMT using an inverse-variance weighted fixed-effect individual participant data meta-analysis (IPD-MA) using the “ipdmetan” package in STATA. This approach offered standardisation of analyses across study sites while taking into account potential clustering and heterogeneity of the different study populations [36]. We were thus able to compare the magnitude of the effect of each adiposity phenotype on CIMT across the various study sites. In these analyses, we obtained a test of the overall effect of each adiposity measure on CIMT in the total sample. We also obtained between-study variance as a percentage of the total variance between study populations (*I^2^*), giving us an idea of the extent of heterogeneity. Forest plots are plotted to display the effect measure and the percentage weight of the various study populations.

Where heterogeneity is significant as demonstrated by a higher % *I^2^*, subgroup analyses were conducted for each site. In these sub-group analyses, the association of adiposity phenotypes with CIMT in each separate site was assessed using adjusted multiple linear regression. These analyses were done in multiple sequential models. In the first step, we initially determined the independent association between each adiposity phenotype and CIMT. We then adjusted for covariates in a sequential manner. In Model 1, we adjusted for age, level of education, and household SES; Model 2 included variables from Model 1 plus additional adjustments for smoking, alcohol consumption, and MVPA. Model 3 was based on Model 2 plus each of the other body adiposity phenotypes. Adiposity phenotypes that had a variance inflation factor (VIF) >5 were dropped from Model 3. Thus, BMI, WC and HC were strongly correlated and were therefore not included in Model 3, whereas VAT and SCAT were included. Model 4 included Model 3 plus systolic blood pressure, glucose, HDL-C, LDL-C, total cholesterol and HIV infection. In women, menopausal status was also adjusted for in Model 4.

Measures of associations are reported as standardised beta (β) coefficients denoting differences in mean CIMT in µm caused by a unit increase in the adiposity phenotypes. Statistical significance was set at two–sided, p < 0.05.

## Results

Data were available for 10,863 participants, but 1,341 had no CIMT data which included all women from Soweto. In addition, 512 participants had missing data from the other variables. Therefore, we conducted complete case analyses of 9,010 participants from six sites in four countries.

### Descriptive data

The basic characteristics of the AWI–Gen study participants stratified by study site and sex are presented in Table 1. For the combined data from all sites, women had a mean age of 50.1±6 years compared to 49.8±9 years for men. In all sites, men had a higher mean household SES compared to women and similar observations were made for educational attainment (Table 1). Men showed a higher prevalence of both current smoking and alcohol consumption than women at all sites. Physical activity levels varied across sites, but women were more likely to be physically active. The prevalence of obesity was higher in women than men at all sites except Nanoro, Burkina Faso. Both women and men from Navrongo had the highest mean common CIMT followed by Nanoro, Dikgale, Agincourt and Soweto (men only) with the lowest levels in both sexes being observed in Nairobi (Table 1).

**Table 1:** Basic characteristics of AWI–Gen study participants from six sites stratified by sex.

Characteristics	All sites (N = 9010)	Nanoro (n = 2074)	Navrongo (n = 1729)	Agincourt (n = 1248)	Dikgale (n = 1142)	Soweto (n = 904)	Nairobi (n = 1913)

**Women (n (%))**	**4536 (50.3)**	**1033 (49.8)**	**935 (54.1)**	**738 (59.1)**	**788 (69.0)**	**–**	**1042 (54.5)**
Age (years)	50.1 ± 5.77	49.79 ± 5.65	51.53 ± 5.74	50.29 ± 5.69	50.53 ± 5.95	–	48.28 ± 5.29
Formal education	2221 (51.5)	70 (6.84)	235 (22.2)	253 (66.1)	723 (90.9)	–	940 (89.4)
Household SES	9.61 ± 3.80	10.74 ± 3.39	9.01 ± 4.52	6.86 ± 2.89	10.07 ± 3.06	–	10.79 ± 3.01
Current alcohol	1383 (32.0)	618 (59.9)	581 (54.7)	135 (18.3)	100 (12.6)	–	294 (28.2)
Current Smoking	176 (3.89)	2 (0.19)	31 (3.32)	9 (1.22)	54 (6.86)	–	80 (7.68)
Obesity	1084 (23.9)	12 (1.16)	36 (3.85)	302 (41.3)	402 (51.0)	–	332 (31.9)
Low MVPA	545 (12.6)	136 (13.2)	199 (18.7)	85 (22.2)	27(3.44)	–	95 (9.12)
Height (m)	1.59 ± 0.07	1.62 ± 0.06	1.58 ± 0.07	1.61 ± 0.07	1.59 ± 0.07	–	1.59 ± 0.06
Weight (kg)	64.25 ± 17.81	53.20 ± 9.42	55.58 ± 10.71	75.67 ± 18.81	77.76 ± 20.76	–	68.48 ± 15.45
Systolic blood pressure (mmHg)	121.7 ± 22.7	111.2 ± 17.5	123.3 ± 23.0	134.5 ± 23.4	126.7 ± 21.1	–	117.8 ± 21.8
Diastolic blood pressure (mmHg)	77.5 ± 12.9	71.1 ± 9.81	77.1 ± 12.6	80.6 ± 13.2	82.5 ± 12.9	–	78.3 ± 13.2
Glucose (mmol/l)	5.09 ± 1.75	5.00 ± 0.91	4.59 ± 0.70	4.94 ± 1.39	5.28 ± 2.46	–	5.59 ± 2.36
LDL-C (mmol/l)	2.27 ± 0.98	1.93 ± 0.81	1.71 ± 0.72	2.34 ± 0.94	2.64 ± 1.02	–	2.86 ± 0.91
HDL-C (mmol/l)	1.15 ± 0.38	1.05 ± 0.35	1.12 ± 0.34	1.19 ± 0.35	1.18 ± 0.35	–	1.26 ± 0.44
HIV+	612 (13.5)	4 (0.39)	6 (0.64)	260 (35.2)	171 (21.7)	–	171 (16.4)
Post-menopausal	1481 (40.1)	352 (34.8)	262 (39.4)	83 (31.8)	376 (52.7)	–	409 (39.1)
Average common CIMT (µm)	636.3 ± 113.9	648.3 ± 112.1	692.2 ± 119.3	609.8 ± 88.9	635.7 ± 111.8	–	593.6 ± 104.9
**Men (n (%))**	**4474 (49.7)**	**1041 (50.2)**	**794 (45.9)**	**510 (40.9)**	**354 (31.0)**	**904 (100)**	**871 (45.5)**
Age (years)	49.8 ± 5.89	49.80 ± 6.00	50.51 ± 5.71	50.25 ± 5.96	50.04 ± 6.01	49.35 ± 5.99	48.78 ± 5.60
Formal education	2959 (67.3)	280 (27.3)	342 (37.9)	252 (77.8)	332 (94.1)	919 (99.1)	834 (96.1)
Household SES	10.62 ± 4.31	11.85 ± 4.18	10.12 ± 4.87	6.52 ± 2.62	9.52 ± 3.38	12.11 ± 3.12	11.59 ± 3.51
Current alcohol	2696 (61.3)	691 (67.3)	702 (77.8)	135 (41.7)	216 (61.2)	660 (71.2)	292 (33.6)
Current Smoking	1521 (34.6)	142 (13.8)	384 (42.5)	81 (25.1)	222 (63.1)	490 (53.0)	202 (23.3)
Obesity	299 (6.69)	23 (2.21)	9 (1.13)	64 (12.6)	10 (2.82)	150 (16.6)	43 (4.94)
Low MVPA	716 (16.3)	255 (24.8)	90 (10.0)	69 (21.3)	13 (3.7)	254 (27.4)	34 (4.0)
Height (m)	1.71 ± 0.07	1.73 ± 0.07	1.67 ± 0.08	1.72 ± 0.07	1.69 ± 0.06	1.71 ± 0.06	1.69 ± 0.07
Weight (kg)	65.91 ± 14.31	65.22 ± 11.98	58.70 ± 9.69	72.59 ± 17.00	62.18 ± 12.62	73.09 ± 17.48	65.58 ± 11.89
Systolic blood pressure (mmHg)	125.4 ± 20.5	120.1 ± 17.8	124.4 ± 20.0	132.2 ± 21.5	124.6 ± 20.4	131.1 ± 21.1	122.4 ± 20.2
Diastolic blood pressure (mmHg)	79.9 ± 13.3	76.0 ± 10.8	76.6 ± 12.9	79.9 ± 12.9	78.4 ± 12.0	89.3 ± 13.2	78.1 ± 2.4
Glucose (mmol/l)	5.05 ± 1.51	5.11 ± 1.51	4.49 ± 0.77	5.06 ± 1.93	4.97 ± 1.71	5.29 ± 1.56	5.24 ± 1.46
LDL-C (mmol/l)	2.36 ± 0.99	2.49 ± 1.01	1.70 ± 0.76	2.29 ± 0.94	2.35 ± 1.01	2.37 ± 0.92	2.87 ± 0.95
HDL-C (mmol/l)	1.22 ± 0.44	1.19 ± 0.38	1.18 ± 0.42	1.21 ± 0.44	1.24 ± 0.47	1.21 ± 0.46	1.28 ± 0.50
HIV+	496 (11.1)	5 (0.48)	7 (0.88)	168 (32.9)	72 (20.3)	179 (19.8)	65 (7.46)
Average common CIMT (µm)	637.9 ± 120	679.9 ± 121	688.1 ± 120	608.2 ± 97	635.7 ± 119	616.9 ± 112	581.9 ± 101

Obesity defined as BMI ≥ 30 kg/m^2^ and data presented as absolute count and percentages = n (%) or as mean ± SD (standard deviation); SES, socio-economic status; MVPA, moderate-to-vigorous physical activity and low is defined as MVPA < 150 mins/week; CIMT, carotid intima-media thickness; LDL-C, low density lipoprotein cholesterol; HDL-C, high density lipoprotein cholesterol and HIV, human immunodeficiency virus.

The mean levels of the various adiposity phenotypes by site and gender are summarized in Table 2. Gender differences (p < 0.001 for all) were noted for all adiposity measures at all sites, with the exception of VAT and SCAT at Nanoro.

**Table 2:** Mean distribution of the various adiposity measures among men and women by study site.

Adiposity phenotype	All sites (N = 9010)	Nanoro (n = 2074)	Navrongo (n = 1729)	Agincourt (n = 1248)	Dikgale (n = 1142)	Soweto (n = 904)	Nairobi (n = 1913)

**Women**							

BMI (kg/m^2^)	25.6 ± 6.99	20.2 ± 3.13	22.1 ± 3.79	29.2 ± 6.75	30.8 ± 7.98	–	27.6 ± 6.08
WC (cm)	85.5 ± 15.2	75.8 ± 8.06	76.3 ± 9.25	94.6 ± 14.9	94.1 ± 16.6	–	90.6 ± 14.3
HC (cm)	98.2 ± 14.4	88.6 ± 7.57	89.1 ± 9.43	105 ± 12.9	109 ± 15.5	–	102 ± 12.1
VAT (cm)	4.94 ± 2.00	4.38 ± 1.16	3.51 ± 1.08	5.74 ± 2.19	6.83 ± 2.25	–	4.77 ± 1.61
SCAT (cm)	1.72 ± 1.14	0.99 ± 0.52	1.13 ± 0.54	2.58 ± 1.74	2.29 ± 1.04	–	1.94 ± 0.71
**Men**							

BMI (kg/m^2^)	22.6 ± 4.56	21.6 ± 3.58	20.9 ± 3.25	24.1 ± 5.33	21.7 ± 3.95	24.9 ± 5.65	22.8 ± 3.89
WC (cm)	82.4 ± 12.5	81.4 ± 9.83	73.2 ± 7.19	87.5 ± 13.5	80.3 ± 11.3	88.9 ± 14.8	83.4 ± 10.7
HC (cm)	91.9 ± 10.6	90.9 ± 9.04	84.1 ± 7.94	95.9 ± 10.2	89.5 ± 8.98	97.9 ± 11.1	93.2 ± 9.65
VAT (cm)	5.33 ± 1.94	4.43 ± 1.32	4.21 ± 1.19	6.47 ± 2.08	6.15 ± 1.87	6.58 ± 2.07	5.21 ± 1.71
SCAT (cm)	1.16 ± 0.89	0.95 ± 0.49	0.77 ± 0.37	1.71 ± 1.79	0.94 ± 0.49	1.55 ± 0.92	1.14 ± 0.54

Data expressed as mean ± SD; BMI, body mass index; WC, waist circumference; HC, hip circumference; VAT, visceral adipose tissue and SCAT, subcutaneous adipose tissue.

### Relationship between adiposity phenotypes and CIMT

**Figure 1:**
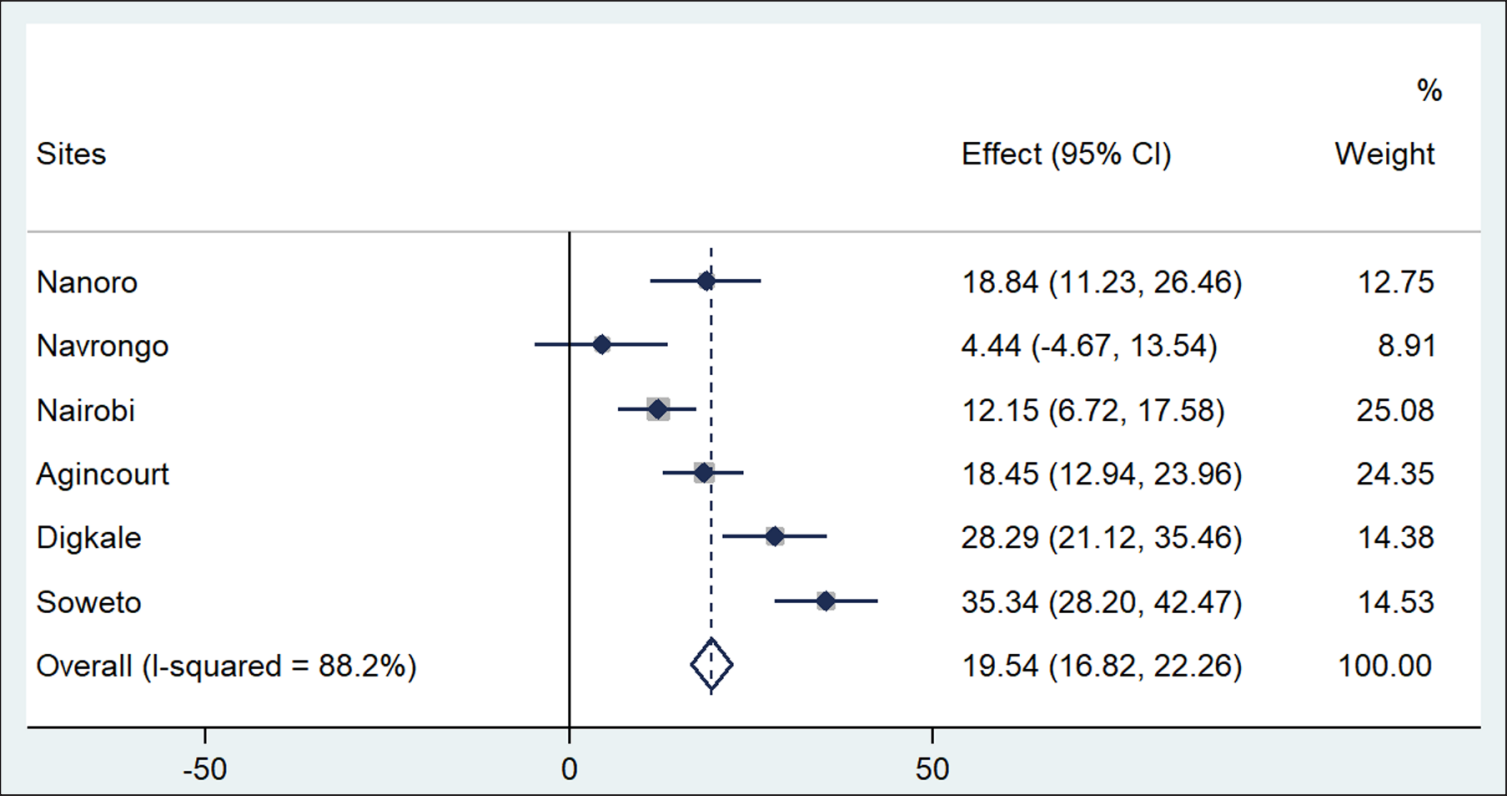
Forest plot displaying an inverse-variance weighted fixed-effect individual participant data meta-analysis of the effect of BMI (kg/m^2^) on common carotid intima-media thickness (µm); the effect size (beta, β) and 95% CIs are presented by the symbol and the bars respectively; the big diamond represents the overall effect of BMI in the poled data and the grey squares represent the % weight of each study site.

**Figure 2:**
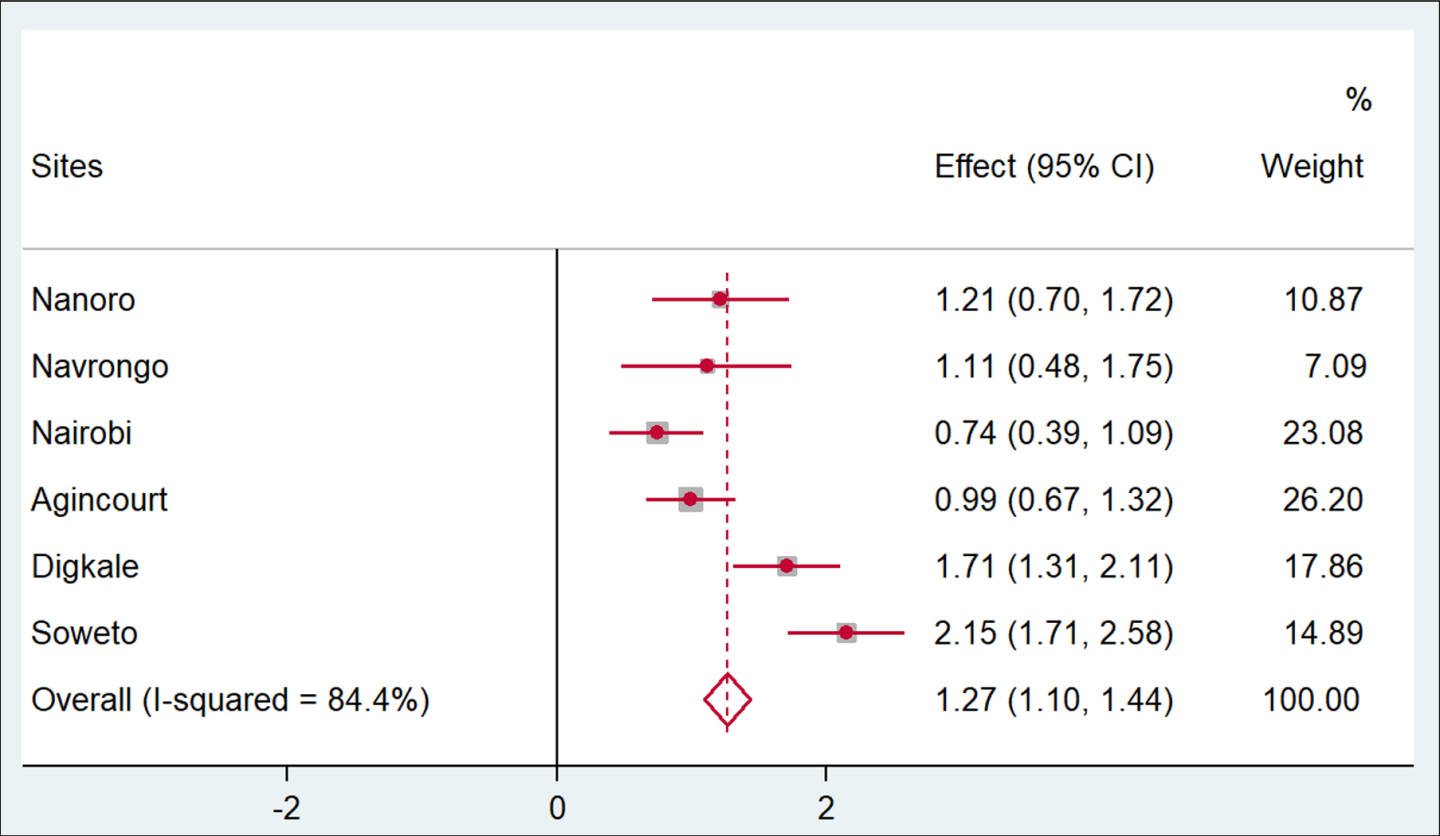
Forest plot displaying an inverse-variance weighted fixed-effect individual participant data meta-analysis of the effect waist circumference (cm) on common carotid intima-media thickness in µm; the effect size (beta, β) and 95% CIs are presented by the symbol and the bars respectively; the big diamond represents the overall effect of waist circumference in the poled data and the grey squares represent the % weight of each study site.

**Figure 3:**
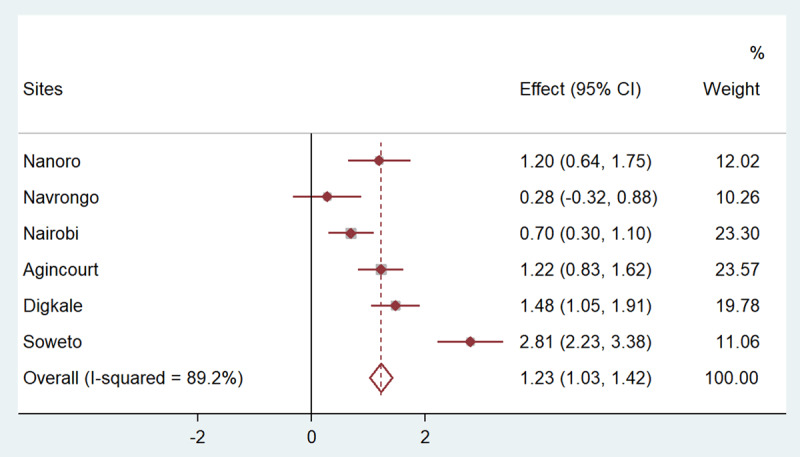
Forest plot displaying an inverse-variance weighted fixed-effect individual participant data meta-analysis of the effect hip circumference (cm) on common carotid intima-media thickness in µm; the effect size (beta, β) and 95% CIs are presented by the symbol and the bars respectively; the big diamond represents the overall effect of hip circumference in the poled data and the grey squares represent the % weight of each study site.

**Figure 4:**
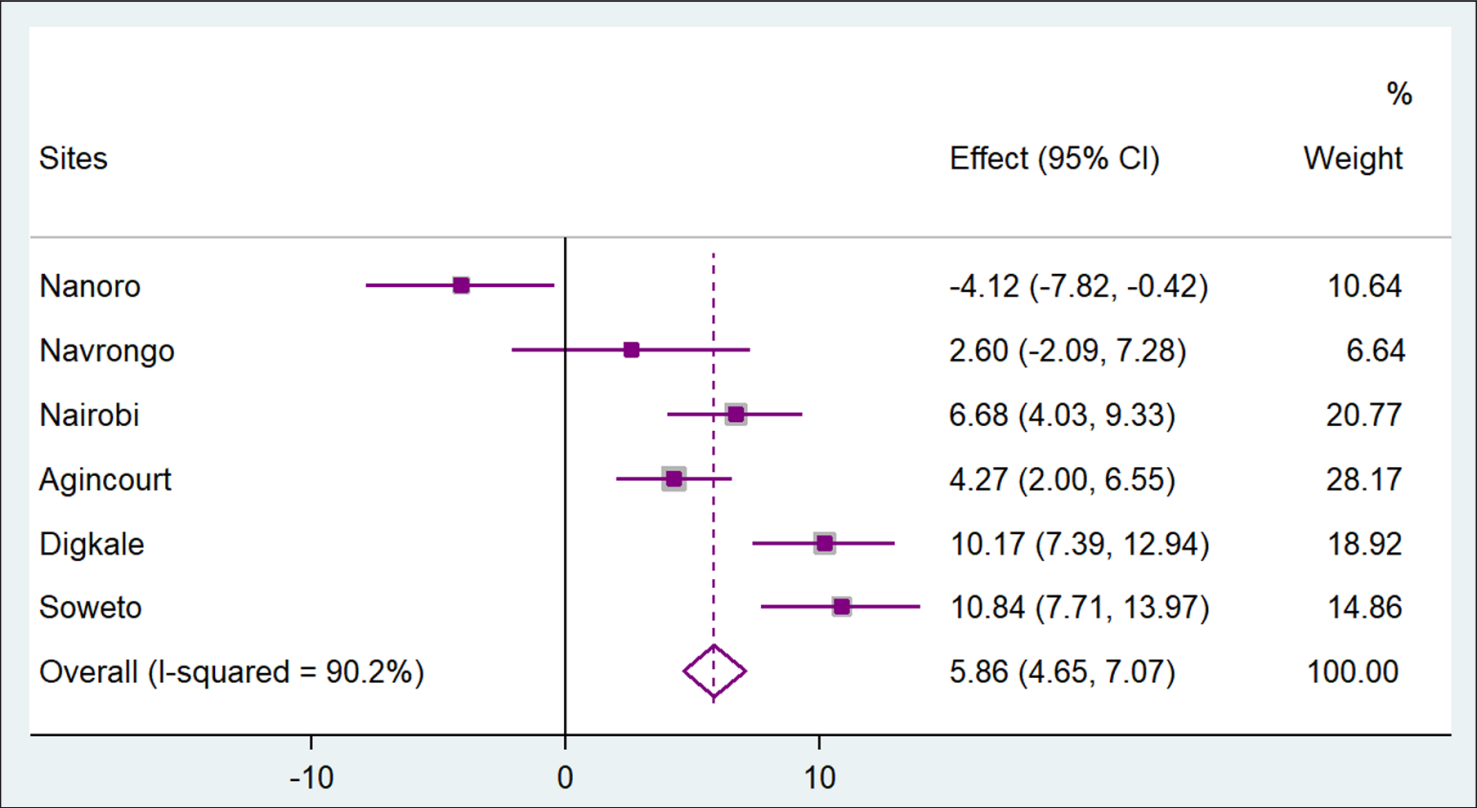
Forest plot displaying an inverse-variance weighted fixed-effect individual participant data meta-analysis of the effect visceral adipose tissue (cm) on common carotid intima-media thickness in µm; the effect size (beta, β) and 95% CIs are presented by the symbol and the bars respectively; the big diamond represents the overall effect of visceral adipose tissue in the pooled data and the grey squares represent the % weight of each study site.

**Figure 5:**
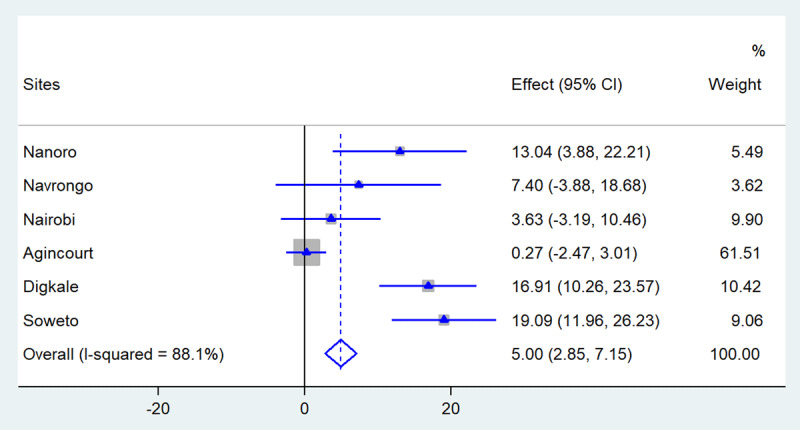
Forest plot displaying an inverse-variance weighted fixed-effect individual participant data meta-analysis of the effect subcutaneous adipose tissue (cm) on common carotid intima-media thickness in µm; the effect size (beta, β) and 95% CIs are presented by the symbol and the bars respectively; the big diamond represents the overall effect of subcutaneous adipose tissue in the pooled data and the grey squares represent the % weight of each study site.

Figures 1, 2, 3, 4, 5 present the forest plots showing the site-specific and pooled IPD-MA of the association of each adiposity phenotype with CIMT. There was an overall positive effect of BMI on CIMT in the pooled analyses and in all sites, but the effect in Navrongo was not statistically significant (Figure 1). The IPD-MA for WC (Figure 2), HC (Figure 3) and SCAT (Figure 5) also gave significant positive pooled effects. In Nanoro, VAT had an inverse association with CIMT while the pooled effect was positive (Figure 4). The pooled IPD-MA demonstrated that BMI had the highest effect measure followed by SCAT, VAT, WC and HC. These analyses demonstrated between-site heterogeneity which shows that the fixed-effect assumption (that the effect is the same at each site) is incorrect. The variability in the effect-size estimates is therefore due to between-site differences rather than sampling variation. We therefore conducted separate analyses for women and men in each site and reported these results in supplementary Tables S1 and S2. The summary measures of heterogeneity and the test of overall effect are presented in supplementary Table S3.

The strength of the associations between each adiposity measures and CIMT stratified by sex varied across the sites, however the direction of the significant associations were the same for all the adiposity phenotypes except VAT and SCAT. VAT was negatively associated with CIMT at Nanoro in both women and men and negatively associated in men at the Navrongo site while SCAT was inversely associated with CIMT among women at all sites. Adjusting for covariates in the models that included all women or all men did tend to affect the strength of the associations, but it did not change the direction of the relationship or render the association significant or non-significant when compared to the initial unadjusted model. The strength of association of each anthropometry measure with CIMT was higher in men than in women at all the study sites (supplementary Tables S1 and S2).

## Discussion

In this study, we examined the association between various adiposity phenotypes and common CIMT in African populations from Burkina Faso, Ghana, Kenya and South Africa. The selected age group of 40–60 years has been identified by World Health Organization to be at risk of “premature death” from CVDs [5], indicating significant public health implications of the current findings. We standardized our estimates to enable us to compare the magnitude of the effect of the various adiposity phenotypes on CIMT by sex and the various study sites. We observed that all adiposity phenotypes were positively associated with an increase in CIMT in the pooled analyses. The associations were stronger in men than in women except for SCAT where the association was stronger in women than in men. We further observed that, after adjusting for possible confounding variables, BMI had the strongest association with CIMT followed by VAT, SCAT, WC and HC.

Our finding that various adiposity phenotypes were associated with greater CIMT in both women and men expands the evidence on this matter since it has been previously reported in European, American, African–American and Asian populations [17,18,19,37,38]. In our study, BMI showed a stronger association with CIMT compared to the other measures of adiposity. This is contrary to many studies which have shown that central adiposity, particularly VAT, is a stronger determinant of CVD risk than BMI [11,39]. However, none of these studies included data collected from African populations resident in sub–Saharan Africa. A study from Malawi previously reported a stronger association of general obesity with hyperglycemia and elevated blood pressure over that of central obesity [40]. In support of the results produced in our investigation, a study conducted on HIV-positive participants from Uganda demonstrated that BMI, but not waist circumference, correlated positively with CIMT [41]. The attenuated effect of VAT and markers of central adiposity on CIMT in sub–Saharan African populations may be related to the lower level of VAT observed in these populations when compared to other ethnic groups [12,13,42,43].

We observed that there was a positive effect of BMI on CIMT in the two West African sites which had the lowest levels of BMI but the highest CIMT levels. Biologically, obesity is known to be associated with CVD risk and our finding supports this observation. However, we noticed the magnitude of the effect of BMI on atherosclerosis was smaller in these two sites compared to the other sites that had higher obesity levels. The high CIMT in West Africa must therefore be due to other variables that were not captured in this study as many factors are known to influence CIMT other than BMI.

The association between various measures of body adiposity and CIMT may be mediated by other CVD risk factors such as high serum cholesterol and blood pressure levels. However, after adjusting for these risk factors in multivariable regression models, the association between adiposity phenotype and CIMT was attenuated, but still remained significant. Thus, other factors must be mediating the effect of adiposity on CIMT and previous studies suggest that these may include inflammation, insulin resistance and endothelial dysfunction [44].

In our study, SCAT was positively associated with CIMT in men but inversely associated with CIMT in women, and these gender–specific associations were consistently observed at each study site. The inverse association of SCAT with CIMT observed in women is supported by other studies that have observed that SCAT may be protective against several diseases including atherosclerosis. Narumi et al (2009), who assessed atherosclerosis using the calcium score of the whole aorta, demonstrated an inverse association between SCAT and atherosclerosis in an apparently healthy Japanese population [45]. Bouchi et al (2015) further observed a protective effect of SCAT on CIMT among Japanese type 2 diabetes patients [46]. A study by Glesby et al (2018) in a group of HIV-positive American females showed that higher SCAT levels were associated with a lower risk of carotid artery plaques [47]. The protective effect of SCAT may be due to the expression of a less pro-inflammatory adipokine profile compared to visceral fat [39,48] and/or the ability of SCAT to act as a buffer for lipid flux thus reducing triglyceride deposition in visceral or ectopic fat depots [49]. Women had higher mean SCAT levels compared to men and this may contribute to the greater protective effect observed in this gender.

In addition, abdominal SCAT is composed of two different compartments termed superficial and deep SCAT, with women having lower levels of deep but higher levels of superficial SCAT than men [50]. Furthermore, a study has shown that deep SCAT is positively associated with the Framingham risk score in multivariable regression models in men but not women [51]. It is uncertain whether these sex differences in abdominal SCAT compartments and their differential association with cardiovascular risk may explain the opposing effects of SCAT on CIMT observed in men and women. Levels of deep and superficial SCAT were not measured in this study and further analysis of these SCAT compartments and their association with CIMT is required in African populations. Also, the higher 95% CIs for the coefficients for SCAT and the high standard deviations for the SCAT mean values may imply some level of measurement imprecision, further affecting the outcome.

We observed that the association of each adiposity phenotype (with the exception of SCAT) with CIMT was stronger in men than women. This is supported by a study from Taiwan which showed that BMI was more strongly associated with CIMT in men than women [52]. Studies have shown that sex differences exist in adipocyte function and secretory profiles [53], but whether such differences explain the differential effects of obesity on CIMT in men and women is not known. It is also possible that this sex difference may be due to differences in the component causes for increased CIMT between men and women.

At most of the study sites, VAT was positively associated with CIMT, but in men in Navrongo and both women and men in Nanoro, West Africa, these associations were in the opposite direction. The reason for this is not known. However, VAT levels at these sites were the lowest of all the sites, whilst CIMT was the highest. In addition, the majority of the participants from West Africa were subsistence farmers, whilst this was not the case in the other regions. It is therefore possible that biological, behavioural and socio-demographic differences across the sites that have not been captured in the current study may explain these variations in the association of VAT with CIMT, and require further investigation. However, it must also be noted that some of the site differences may be due to random error or differences in the population selection at the various sites.

### Strengths and limitations

This study contains data on CIMT and adiposity phenotypes from a large population of black African subjects from three sub-continental regions, that is, East, West and South Africa and, to the best of our knowledge, is the largest study conducted on populations residing in SSA. Measurements of exposure and outcome variables and covariates were harmonized across the six study sites. This minimized variability and enabled us to make valid comparisons by sex and site across the study populations. Most large population-based studies use crude markers of adiposity, however, in this study we used ultrasound to assess VAT and SCAT levels. Due to the broad range of adiposity measures used in our analyses, we were able to investigate the contribution of general, central and peripheral adiposity to atherosclerosis in the study population. Furthermore, we adjusted for a wide range of confounding variables including socio–economic status, lifestyle factors and other cardiovascular risk factors, to minimize the effect of residual confounding.

There are some limitations to our study. Due to the cross–sectional study design, it is not possible to infer causality. Even though we included a wide range of possible confounding variables, our observed associations may still be affected by persistent residual confounding due to unmeasured variables that may better explain reported associations. Since most of the risk factors were self–reported, there is the risk of recall bias and selective response leading to some biased estimates. The gold–standard methods for measurement of VAT and SCAT include CT and MRI imaging, whereas we used B–mode ultrasound measures. However, this method was the only practical way of measuring VAT, SCAT and CIMT in a large population–based study across multiple sites. Despite significant efforts instituted to reduce inter-sonographer measurement variation, we fully acknowledge that there may be some level of error obtained in the measurements in the field.

## Conclusions

The results from this study suggest that the increasing prevalence of obesity within SSA will likely result in a surge in atherosclerotic cardio- and cerebrovascular events, especially in males, unless addressed through primary prevention of obesity in both rural and urban communities across Africa. The inverse association between VAT and CIMT in West Africa (Burkina Faso and Ghana) requires further investigation.

## Additional File

The additional file for this article can be found as follows:

10.5334/gh.863.s1 Supplemental Material. Supplementary material contains site specific multiple linear regression analyses with sequential model building approach (Table S1 and S2) and summary measures of heterogeneity from the individual participant data meta-analyses (IPD-MA).

## References

[1] Roth GA, Johnson C, Abajobir A, Abd-Allah F, Abera SF, Abyu G, et al. Global, regional, and national burden of cardiovascular diseases for 10 causes, 1990–2015. J Am Coll Cardiol. 2017; 70(1): 1–25. DOI: 10.1016/j.jacc.2017.04.052 28527533 PMC5491406

[2] NCD Risk Factor Collaboration (NCD-RisC) – Africa Working Group. Trends in obesity and diabetes across Africa from 1980–2014: An analysis of pooled population-based studies. Int J Epidemiol. 2017; 46(5): 1421–32. 28582528 10.1093/ije/dyx078 PMC5837192

[3] Ramsay M, Crowther NJ, Agongo G, Ali SA, Asiki G, Boua RP, et al. Regional and sex-specific variation in BMI distribution in four sub-Saharan African countries: The H3Africa AWI-Gen study. Glob Health Action. 2018; 11(sup2): 1556561. DOI: 10.1080/16549716.2018.1556561 30845902 PMC6407581

[4] Yusuf S, Reddy S, Ôunpuu S, Anand S. Global burden of cardiovascular diseases part I: General considerations, the epidemiologic transition, risk factors, and impact of urbanization. Circulation. 2001; 104: 2746–53. DOI: 10.1161/hc4601.099487 11723030

[5] GBD 2015 Risk Factors Collaborators. Global, regional, and national comparative risk assessment of 79 behavioural, environmental and occupational, and metabolic risks or clusters of risks, 1990–2015: A systematic analysis for the Global Burden of Disease Study 2015. Lancet. 2016; 388(10053): 1659–724. DOI: 10.1016/S0140-6736(16)31679-8 27733284 PMC5388856

[6] NCD Risk Factor Collaboration (NCD-RisC). Rising rural body-mass index is the main driver of the global obesity epidemic in adults. Nature. 2019; 569(7755): 260–4. DOI: 10.1038/s41586-019-1171-x 31068725 PMC6784868

[7] Libby P. Inflammation and atherosclerosis. Circulation. 2002; 105(9): 1135–43. DOI: 10.1161/hc0902.104353 11877368

[8] Kinlen D, Cody D, O’Shea D. Complications of obesity. QJM. 2018; 111(7): 437–43. DOI: 10.1093/qjmed/hcx152 29025162

[9] Wang H, Chen YE, Eitzman DT. Imaging body fat: Techniques and cardiometabolic implications. Arterioscler Thromb Vasc Biol. 2014; 34(10): 2217–23. DOI: 10.1161/ATVBAHA.114.303036 25147343 PMC4169325

[10] Cornier MA, Despres JP, Davis N, Grossniklaus DA, Klein S, Lamarche B, et al. Assessing adiposity: A scientific statement from the American Heart Association. Circulation. 2011; 124(18): 1996–2019. DOI: 10.1161/CIR.0b013e318233bc6a 21947291

[11] Lee MJ, Wu Y, Fried SK. Adipose tissue heterogeneity: implication of depot differences in adipose tissue for obesity complications. Mol Aspects Med. 2013; 34(1): 1–11. DOI: 10.1016/j.mam.2012.10.001 23068073 PMC3549425

[12] Goedecke JH, Levitt NS, Lambert EV, Utzschneider KM, Faulenbach MV, Dave JA, et al. Differential effects of abdominal adipose tissue distribution on insulin sensitivity in black and white South African women. Obesity (Silver Spring). 2009; 17(8): 1506–12. DOI: 10.1038/oby.2009.73 19300428

[13] Sumner AE, Micklesfield LK, Ricks M, Tambay AV, Avila NA, Thomas F, et al. Waist circumference, BMI, and visceral adipose tissue in white women and women of African descent. Obesity (Silver Spring). 2011; 19(3): 671–4. DOI: 10.1038/oby.2010.201 20847732 PMC3474331

[14] Mancia G, Fagard R, Narkiewicz K, Redon J, Zanchetti A, Bohm M, et al. 2013 ESH/ESC Guidelines for the management of arterial hypertension: The Task Force for the management of arterial hypertension of the European Society of Hypertension (ESH) and of the European Society of Cardiology (ESC). J Hypertens. 2013; 31(7): 1281–357. DOI: 10.1097/01.hjh.0000431740.32696.cc 23817082

[15] O’Leary DH, Bots ML. Imaging of atherosclerosis: Carotid intima–media thickness. Eur Heart J. 2010; 31(14): 1682–9. DOI: 10.1093/eurheartj/ehq185 20542989

[16] Ruijter HMD, Peters SAE, Anderson TJ, Britton AR, Dekker JM, Eijkemans MJ, et al. Common carotid intima-media thickness measurements in cardiovascular risk prediction: A meta-analysis. JAMA. 2012; 308(8): 796–803. DOI: 10.1001/jama.2012.9630 22910757

[17] Chen LY, Leening MJ, Norby FL, Roetker NS, Hofman A, Franco OH, et al. Carotid intima-media thickness and arterial stiffness and the risk of atrial fibrillation: The Atherosclerosis Risk in Communities (ARIC) study, Multi–Ethnic Study of Atherosclerosis (MESA), and the Rotterdam Study. J Am Heart Assoc. 2016; 5(5). DOI: 10.1161/JAHA.115.002907 PMC4889172 27207996

[18] Jin-Kee P, Hyuntae P, Kwi-Baek K. The relationship between distribution of body fat mass and carotid artery intima-media thickness in Korean older adults. J Phys Ther Sci. 2015; 27(10): 3141–6. DOI: 10.1589/jpts.10.3141 26633917 PMC4666713

[19] Kawamoto R, Ohtsuka N, Ninomiya D, Nakamura S. Association of obesity and visceral fat distribution with intima-media thickness of carotid arteries in middle-aged and older persons. Internal Medicine. 2008; 47(3): 143–9. DOI: 10.2169/internalmedicine.47.0478 18239322

[20] Oike M, Yokokawa H, Fukuda H, Haniu T, Oka F, Hisaoka T, et al. Association between abdominal fat distribution and atherosclerotic changes in the carotid artery. Obes Res Clin Pract. 2014; 8(5): e448–58. DOI: 10.1016/j.orcp.2013.09.002 25263834

[21] Lin A, Lacy ME, Eaton C, Correa A, Wu W-C. Inflammatory obesity phenotypes, gender effects and subclinical atherosclerosis in African Americans: The Jackson Heart Study. Arterioscler Thromb Vasc Biol. 2016; 36(12): 2431–8. DOI: 10.1161/ATVBAHA.116.307728 27856456 PMC5121048

[22] Ramsay M, Sankoh O, as members of the AWIGen study and the H3Africa Consortium. African partnerships through the H3Africa Consortium bring a genomic dimension to longitudinal population studies on the continent. Int J Epidemiol. 2016; 45(2): 305–8. DOI: 10.1093/ije/dyv187 26659658 PMC5841636

[23] Ramsay M, Crowther NJ, Tambo E, Agongo G, Baloyi V, Dikotope S, et al. H3Africa AWI-Gen collaborative centre: a resource to study the interplay between genomic and environmental risk factors for cardiometabolic diseases in four sub-Saharan African countries. Global Health, Epidemiology and Genomics. 2016; 1(e20): 1–13. DOI: 10.1017/gheg.2016.17 PMC5732578 29276616

[24] Ali SA, Soo C, Agongo G, Alberts M, Amenga-Etego L, Boua RP, et al. Genomic and environmental risk factors for cardiometabolic diseases in Africa: methods used for Phase 1 of the AWI-Gen population cross-sectional study. Glob Health Action. 2018; 11(sup2): 1507133. DOI: 10.1080/16549716.2018.1507133 30259792 PMC6161608

[25] Richter L, Norris S, Pettifor J, Yach D, Cameron N. Cohort profile: Mandela’s children: The 1990 Birth to twenty study in South Africa. Int J Epidemiol. 2007; 36(3): 504–11. DOI: 10.1093/ije/dym016 17355979 PMC2702039

[26] Gomez-Olive FX, Montana L, Wagner RG, Kabudula CW, Rohr JK, Kahn K, et al. Cohort Profile: Health and ageing in Africa: A longitudinal study of an INDEPTH community in South Africa (HAALSI). Int J Epidemiol. 2018 (1464–3685 (Electronic)). DOI: 10.1093/ije/dyx247 PMC6005147 29325152

[27] Alberts M, Dikotope SA, Choma SR, Masemola ML, Modjadji SEP, Mashinya F, et al. Health & demographic surveillance system profile: The dikgale health and demographic surveillance system. International Journal of Epidemiology. 2015; 44(5): 1565–71. DOI: 10.1093/ije/dyv157 26275454

[28] Beguy D, Elung’ata P, Mberu B, Oduor C, Wamukoya M, Nganyi B, et al. Health & demographic surveillance system rofile: The Nairobi Urban Health and Demographic Surveillance System (NUHDSS). Int J Epidemiol. 2015; 44(2): 462–71. DOI: 10.1093/ije/dyu251 25596586

[29] Derra K, Rouamba E, Kazienga A, Ouedraogo S, Tahita MC, Sorgho H, et al. Profile: Nanoro health and demographic surveillance system. Int J Epidemiol. 2012; 41(5): 1293–301. DOI: 10.1093/ije/dys159 23045201

[30] Oduro AR, Wak G, Azongo D, Debpuur C, Wontuo P, Kondayire F, et al. Profile of the navrongo health and demographic surveillance system. Int J Epidemiol. 2012; 41(4): 968–76. DOI: 10.1093/ije/dys111 22933645

[31] Dogan S, Plantinga Y, Crouse JR, Evans GW, Raichlen JS, O’Leary DH, et al. Algorithms to measure carotid intima-media thickness in trials: A comparison of reproducibility, rate of progression and treatment effect. J Hypertens. 2011; 29(11): 2181–93. DOI: 10.1097/HJH.0b013e32834b0eba 21918474

[32] Touboul PJ, Hennerici MG, Meairs S, Adams H, Amarenco P, Bornstein N, et al. Mannheim carotid intima-media thickness and plaque consensus (2004–2006–2011). An update on behalf of the advisory board of the 3rd, 4th and 5th watching the risk symposia, at the 13th, 15th and 20th European Stroke Conferences, Mannheim, Germany, 2004, Brussels, Belgium, 2006, and Hamburg, Germany, 2011. Cerebrovasc Dis. 2012; 34(4): 290–6. DOI: 10.1159/000343145 23128470 PMC3760791

[33] Nambi V, Chambless L, He M, Folsom AR, Mosley T, Boerwinkle E, et al. Common carotid artery intima-media thickness is as good as carotid intima-media thickness of all carotid artery segments in improving prediction of coronary heart disease risk in the Atherosclerosis Risk in Communities (ARIC) study. Eur Heart J. 2012; 33(2): 183–90. DOI: 10.1093/eurheartj/ehr192 21666250 PMC3258447

[34] Stolk RP, Meijer R, Mali WP, Grobbee DE, van der Graaf Y. Ultrasound measurements of intraabdominal fat estimate the metabolic syndrome better than do measurements of waist circumference. Am J Clin Nutr. 2003; 77(4): 857–60. DOI: 10.1093/ajcn/77.4.857 12663283

[35] De Lucia Rolfe E, Norris SA, Sleigh A, Brage S, Dunger DB, Stolk RP, et al. Validation of ultrasound estimates of visceral fat in black South African adolescents. Obesity. 2011; 19(9): 1892–7. DOI: 10.1038/oby.2011.213 21738240

[36] Riley RD, Lambert PC, Abo-Zaid G. Meta-analysis of individual participant data: Rationale, conduct, and reporting. BMJ. 2010; 340: c221. DOI: 10.1136/bmj.c221 20139215

[37] Gast KB, den Heijer M, Smit JW, Widya RL, Lamb HJ, de Roos A, et al. Individual contributions of visceral fat and total body fat to subclinical atherosclerosis: The NEO study. Atherosclerosis. 2015; 241(2): 547–54. DOI: 10.1016/j.atherosclerosis.2015.05.026 26100677

[38] Wildman RP, Janssen I, Khan UI, Thurston R, Barinas-Mitchell E, El Khoudary SR, et al. Subcutaneous adipose tissue in relation to subclinical atherosclerosis and cardiometabolic risk factors in midlife women. Am J Clin Nutr. 2011; 93(4): 719–26. DOI: 10.3945/ajcn.110.007153 21346089 PMC3057544

[39] Fantuzzi G, Mazzone T. Adipose tissue and atherosclerosis: exploring the connection. Arterioscler Thromb Vasc Biol. 2007; 27(5): 996–1003. DOI: 10.1161/ATVBAHA.106.131755 17303782

[40] Mudie K, Lawlor DA, Pearce N, Crampin A, Tomlinson L, Tafatatha T, et al. How does the association of general and central adiposity with glycaemia and blood pressure differ by gender and area of residence in a Malawian population: A cross-sectional study. Int J Epidemiol. 2018; 47(3): 887–98. DOI: 10.1093/ije/dyy047 29648664 PMC6005143

[41] Sinabulya I, Kayima J, Longenecker C, Luwedde M, Semitala F, Kambugu A, et al. Subclinical atherosclerosis among HIV-infected adults attending HIV/AIDS care at two large ambulatory HIV clinics in Uganda. PLoS One. 2014; 9(2): e89537. DOI: 10.1371/journal.pone.0089537 24586854 PMC3938501

[42] van der Merwe MT, Crowther NJ, Schlaphoff GP, Gray IP, Joffe BI, Lonnroth PN. Evidence for insulin resistance in black women from South Africa. Int J Obes Relat Metab Disord. 2000; 24(10): 1340–6. DOI: 10.1038/sj.ijo.0801416 11093297

[43] Waisberg R, Paiker JE, Crowther NJ. Adipokine serum concentrations, anthropometric measurements and socio-economic status in two ethnic groups with different prevalence levels for cardiovascular diseases and type 2 diabetes. Horm Metab Res. 2011; 43(9): 660–6. DOI: 10.1055/s-0031-1283139 21823063

[44] Lovren F, Teoh H, Verma S. Obesity and atherosclerosis: Mechanistic insights. Can J Cardiol. 2015; 31(2): 177–83. DOI: 10.1016/j.cjca.2014.11.031 25661552

[45] Narumi H, Yoshida K, Hashimoto N, Umehara I, Funabashi N, Yoshida S, et al. Increased subcutaneous fat accumulation has a protective role against subclinical atherosclerosis in asymptomatic subjects undergoing general health screening. International Journal of Cardiology. 2009; 135(2): 150–5. DOI: 10.1016/j.ijcard.2008.03.044 18593641

[46] Bouchi R, Takeuchi T, Akihisa M, Ohara N, Nakano Y, Nishitani R, et al. High visceral fat with low subcutaneous fat accumulation as a determinant of atherosclerosis in patients with type 2 diabetes. Cardiovasc Diabetol. 2015; 14: 136. DOI: 10.1186/s12933-015-0302-4 26445876 PMC4597374

[47] Chen P, Hou X, Hu G, Wei L, Jiao L, Wang H, et al. Abdominal subcutaneous adipose tissue: a favorable adipose depot for diabetes? Cardiovasc Diabetol. 2018; 17(1): 93. DOI: 10.1186/s12933-018-0734-8 29945626 PMC6020307

[48] Porter SA, Massaro JM, Hoffmann U, Vasan RS, O’Donnel CJ, Fox CS. Abdominal subcutaneous adipose tissue: a protective fat depot? Diabetes Care. 2009; 32(6): 1068–75. DOI: 10.2337/dc08-2280 19244087 PMC2681034

[49] Frayn KN. Adipose tissue as a buffer for daily lipid flux. Diabetologia. 2002; 45(9): 1201–10. DOI: 10.1007/s00125-002-0873-y 12242452

[50] Kohli S, Sniderman AD, Tchernof A, Lear SA. Ethnic-specific differences in abdominal subcutaneous adipose tissue compartments. Obesity (Silver Spring). 2010; 18(11): 2177–83. DOI: 10.1038/oby.2010.94 20448537

[51] Marinou K, Hodson L, Vasan SK, Fielding BA, Banerjee R, Brismar K, et al. Structural and functional properties of deep abdominal subcutaneous adipose tissue explain its association with insulin resistance and cardiovascular risk in men. Diabetes Care. 2014; 37(3): 821–9. DOI: 10.2337/dc13-1353 24186879

[52] Wu TW, Hung CL, Liu CC, Wu YJ, Wang LY, Yeh HI. Associations of cardiovascular risk factors with carotid intima-media thickness in middle-age adults and elders. J Atheroscler Thromb. 2017; 24(7): 677–86. DOI: 10.5551/jat.37895 27874838 PMC5517541

[53] Chang E, Varghese M, Singer K. Gender and sex differences in adipose tissue. Curr Diab Rep. 2018; 18(9): 69. DOI: 10.1007/s11892-018-1031-3 30058013 PMC6525964

