# Supplementary material for: Adiposity Phenotypes and Subclinical Atherosclerosis in Adults from Sub–Saharan Africa: An H3Africa AWI–Gen Study
Source: Glob Heart. 2021 Mar 19;16(1):19. doi: 10.5334/gh.863 (PMC7977036; doi:10.5334/gh.863)
Supplement: Supplemental Material. — Supplementary material contains site specific multiple linear regression analyses with sequential model building approach (Table S1 and S2) and summary measures of heterogeneity from the individual participant data meta-analyses (IPD-MA). [file gh-16-1-863-s1.pdf]

Table S1: Association of adiposity phenotypes with common CIMT in women aged 40-60 years from SSA stratified by site

	Nanoro, Burkina Faso	Navrongo, Ghana	Agincourt, South Africa	Dikgale , South Africa	Soweto, South Africa	Nairobi, Kenya	Combined population
	Standardised β (95%CI)	Standardised β (95%CI)	Standardised β (95%CI)	Standardised β (95%CI)	-	Standardised β (95%CI)	Standardised β (95%CI)
**BMI (SD=6.9kg/m^{2})**							
Crude model	0.59 (-1.43, 2.63)	-1.17 (-3.25, 0.92)	1.77 (0.89, 2.65)	2.53 (1.54, 3.52)	-	1.34 (0.30, 2.38)	0.91 (0.41, 1.41)
Model 1	1.94 (-0.13, 4.02)	1.06 (-1.12, 3.25)	1.49 (0.63, 2.36)	2.46 (1.50, 3.41)	-	1.04 (-0.01, 2.09)	0.85 (0.35, 1.36)
Model 2	1.93 (-0.15, 4.02)	1.09 (-1.13, 3.32)	1.52 (0.66, 2.37)	2.45 (1.49, 3.41)	-	1.04 (-0.01, 2.09)	0.96 (0.45, 1.46)
Model 3	5.56 (2.76, 8.36)	0.37 (-2.59, 3.34)	1.69 (0.37, 3.03)	1.97 (0.15, 3.79)	-	1.80(0.28, 3.33)	2.68 (1.89, 3.47)
Model 4	4.97 (2.13, 7.81)	-0.32 (-3.25, 2.60)	1.54 (0.19, 2.89)	2.08 (0.23, 3.93)	-	1.74 (0.09, 3.41)	2.30 (1.49, 3.11)
**WC (SD=15.2cm)**							
Crude model	0.68 (-0.12, 1.48)	0.51 (-0.33, 1.35)	0.84 (0.48, 1.21)	1.44 (0.98, 1.89)	-	0.75 (0.31, 1.21)	0.46 (0.24, 0.68)
Model 1	0.33 (0.45, 1.12)	0.98 (0.13, 1.84)	0.53 (0.17, 0.89)	1.33 (0.88, 1.78)	-	0.36 (-0.08, 0.80)	0.21 (-0.01, 0.43)
Model 2	0.36 (-0.42, 1.16)	1.04 (0.17, 1.90)	0.54 (0.18, 0.90)	1.31 (0.86, 1.76)	-	0.35 (-0.08, 0.80)	0.25 (0.03, 0.48)
Model 3	1.17 (0.16, 2.18)	2.30 (0.76, 3.85)	0.57 (-0.01, 1.14)	0.81 (0.08, 1.55)	-	0.74 (0.07, 1.41)	0.74 (0.40, 1.08)
Model 4	1.11 (0.06, 2.17)	1.18 (-0.07, 2.42)	0.48 (-0.16, 1.13)	0.97 (0.12, 1.82)		0.69 (-0.03, 1.41)	0.59 (0.23, 0.95)
**HC (SD=14.4cm)**							
Crude model	-0.07 (-0.91, 0.77)	-0.79 (-1.59, 0.02)	0.78 (0.35, 1.22)	1.05 (0.50, 1.60)	-	0.58 (0.07, 1.10)	0.10 (-0.15, 0.35)
Model 1	0.43 (-0.39, 1.25)	-0.06 (-0.85, 0.73)	0.74 (0.32, 1.17)	1.22 (0.73, 1.72)	-	0.44 (-0.06, 0.93)	0.16 (-0.09, 0.41)
Model 2	0.42 (-0.40, 1.25)	-0.05 (-0.86, 0.76)	0.76 (0.34, 1.18)	1.21 (0.71, 1.72)	-	0.43 (-0.06, 0.93)	0.21 (-0.04, 0.46)
Model 3	0.92 (-0.87, 0.93)	-0.44 (-1.36, 0.49)	0.73 (0.16, 1.31)	0.62 (-0.14, 1.38)	-	0.79 (0.10, 1.48)	0.55 (0.19, 0.91)
Model 4	1.01 (-0.03, 2.05)	-0.41 (-1.32, 0.50)	0.77 (0.14, 1.40)	0.52 (-0.31, 1.35)	-	0.91 (0.16, 1.66)	0.44 (0.08, 0.79)
**VAT (SD=23.9cm)**							
Crude model	-6.58 (-12.4, -0.75)	5.26 (-2.14, 12.7)	4.49 (1.72, 7.26)	9.87 (6.52, 13.2)	-	7.62 (3.55, 11.7)	1.71 (0.06, 3.36)
Model 1	-7.47 (-12.9, -1.99)	6.78 (-0.38, 13.9)	4.42 (1.83, 7.02)	8.26 (5.18, 11.3)	-	6.16 (2.33, 9.99)	1.02 (-0.58, 2.62)
Model 2	-7.56 (-13.1, -2.06)	6.55 (-0.56, 13.7)	4.48 (1.86, 7.11)	8.27 (5.17, 11.4)	-	6.23 (2.40, 10.1)	1.18 (-0.42, 2.78)
Model 3	-7.57 (-13.1, -2.05)	7.74 (-0.34, 15.8)	4.45 (1.76, 7.13)	7.04 (2.79, 11.3)	-	8.00 (3.94, 12.07)	1.82(0.19, 3.44)
Model 4	-7.59 (-13.2, -2.01)	5.34 (-2.66, 13.3)	3.02 (0.19, 5.85)	7.01 (2.78, 11.2)	-	5.72 (1.51, 9.94)	2.08 (0.37, 1.65)
**SCAT (SD=7.5cm)**							

Crude model	4.61 (-15.6, 10.4)	-2.38 (-16.4, 11.6)	-0.49 (-3.49, 2.95)	10.4 (2.86, 17.9)	-	-2.39 (-11.0, 6.25)	-2.82 (-5.59, -0.05)
Model 1	-2.59 (-5.19, 3.83)	-2.67 (-11.3, 16.7)	-2.51 (-5.85, -0.84)	13.7 (6.92, 20.55)	-	-3.69 (-12.1, 4.74)	-4.49 (-7.25, -1.74)
Model 2	-2.49 (-15.5, 10.5)	-2.32 (-11.9, 16.6)	-2.52 (-5.90, 0.86)	13.7 (6.86, 20.5)	-	-3.71 (-12.2, 4.77)	-4.13 (-6.91, -1.35)
Model 3	-25.6 (-33.3, 2.07)	-17.6 (-37.7, 2.40)	-3.19 (-8.05, 1.67)	-2.61 (-14.6, 9.35)	-	-21.8 (-34.3, -9.33)	-4.42 (-7.27, -1.57)
Model 4	-6.58 (-20, 7.29)	-12.8 (-29.3, 3.73)	-0.09 (-3.99, 3.80)	-1.25 (-10.6, 8.13)	-	-15.4 (-24.5, -6.32)	-3.94 (-7.03, -0.84)

Model 1: age, educational status and household SES; **Model 2:** Model 1 + alcohol, smoking and physical activity and **Model 3:** model 2 + other adiposity measures except those that were collinear. **Model 4:** Model 3 + glucose, HDL-C, LDL-C, SBP and HIV

Table S2: Association of adiposity phenotypes with CIMT in men aged 40-60 years from SSA stratified by site

	**Nanoro, Burkina Faso**	**Navrongo, Ghana**	**Agincourt, South Africa**	**Dikgale , South Africa**	**Soweto, South Africa**	**Nairobi, Kenya**	**Combined population**
	**Standardised β (95%CI)**	**Standardised β (95%CI)**	**Standardised β (95%CI)**	**Standardised β (95%CI)**	**Standardised β (95%CI)**	**Standardised β (95%CI)**	**Standardised β (95%CI)**
**BMI (SD=4.6kg/m^2)**							
Crude model	3.15 (1.04, 5.25)	-0.39 (-3.10, 2.32)	4.79 (2.90, 6.69)	9.78 (6.24, 13.31)	6.86 (5.48, 8.24)	4.94 (3.17, 6.72)	4.33 (3.51, 5.15)
Model 1	6.54 (4.46, 8.63)	2.64 (-0.30, 5.59)	4.59 (2.82, 6.35)	9.24 (5.88, 12.59)	6.06 (4.79, 7.33)	4.65 (2.94, 6.37)	4.29 (3.50, 5.09)
Model 2	6.17 (4.05, 8.29)	2.82 (-0.19,5.83)	4.79 (2.95, 6.63)	9.18 (5.79, 12.56)	6.30 (4.99, 7.61)	4.64 (2.89, 6.38)	4.49 (3.68, 5.31)
Model 3	6.62 (3.19, 10.1)	2.06 (-1.24, 5.38)	3.22 (6.15, 6.28)	9.92 (4.17, 17.68)	6.01 (2.83, 9.09)	5.54 (2.34, 8.74)	5.44 (4.26, 6.61)
Model 4	6.60 (3.32, 9.88)	2.05 (-0.98, 5.09)	3.41 (0.91, 5.91)	8.69 (3.38, 14.00)	7.16 (4.92, 9.41)	4.87 (2.03, 7.71)	4.86 (3.64, 6.09)
**WC (SD=12.5cm)**							
Crude model	1.38 (0.60, 2.16)	0.99 (-0.27, 2.25)	2.13 (1.41, 2.86)	4.10 (2.81, 5.39)	2.71 (2.19, 3.22)	1.71 (1.08, 2.35)	1.61 (1.31, 1.90)
Model 1	1.98 (1.23, 2.73)	1.47 (0.22, 2.72)	1.80 (1.09, 2.52)	3.52 (2.31, 4.73)	2.18 (1.70, 2.67)	1.48 (0.89, 2.08)	1.30 (1.01, 1.59)
Model 2	1.87 (1.12, 2.61)	1.54(0.28, 2.81)	1.86 (1.13, 2.59)	3.57 (2.35, 4.79)	2.26 (1.77, 2.76)	1.46 (0.86, 2.06)	1.37 (1.07, 1.67)
Model 3	2.34 (1.26, 3.42)	1.57 (0.05, 3.10)	2.08 (1.06, 3.11)	4.72 (2.07, 7.38)	2.33 (1.61, 3.06)	1.27 (0.29, 2.24)	1.62 (1.19, 2.04)
Model 4	2.16 (0.92, 3.39)	1.64 (0.11, 2.79)	1.61 (0.43, 2.79)	3.56 (1.76, 5.36)	2.53 (1.65, 3.41)	1.27 (0.15, 2.39)	1.38 (0.91, 1.84)
**HC (SD=10.6cm)**							
Crude model	0.88(0.01, 1.74)	-0.29(-1.31,0.73)	2.41(1.46,3.36)	3.46(1.96,4.95)	3.35(2.69,4.02)	1.23(0.31,2.16)	1.26 (0.88, 1.63)
Model 1	1.85(0.90, 2.81)	0.86(-0.19,1.92)	2.19(1.31,3.09)	3.29(1.86,4.73)	2.91(2.29,3.52)	1.17(0.18,2.15)	1.22 (0.85, 1.59)
Model 2	1.70(0.74, 2.67)	0.94(-0.12,2.01)	2.25(1.35,3.14)	3.22(1.80,4.65)	3.01(2.38,3.64)	1.12(0.14,2.12)	1.27 (0.89, 1.65)
Model 3	1.43(0.34, 2.52)	0.72(-0.36,1.80)	2.16(1.13,3.19)	2.53(0.87,4.18)	2.70 (1.89,3.51)	0.52(-0.73,1.77)	0.97 (0.49, 1.45)
Model 4	1.55 (0.40, 2.70)	0.56 (-0.54, 1.67)	1.62 (0.42, 2.81)	1.89 (0.07, 3.72)	2.71(1.75, 3.67)	0.36 (-0.94, 1.67)	0.75 (0.25, 1.24)
**VAT (SD=24.6cm)**							
Crude model	-0.61(-6.69, 5.48)	-3.19(-10.4, 4.01)	3.06 (-1.79, 7.91)	14.7(7.37, 21.9)	13.3 (9.41, 17.2)	8.09(4.32, 11.9)	4.72 (2.73, 6.71)
Model 1	-3.10(-8.59, 2.39)	-1.48 (-8.23, 5.27)	3.69 (-0.97, 8.36)	14.7 (7.99, 21.4)	10.6 (6.87, 14.3)	7.26 (3.68, 10.8)	3.99 (2.09, 5.90)
Model 2	-2.15(-7.61, 3.31)	-1.77(-8.52, 4.99)	3.59(-1.05, 8.24)	14.4(7.81, 21.0)	10.9(7.16, 14.8)	7.34(3.77, 10.9)	4.09 (2.19, 5.98)
Model 3	-5.21(-11.1, 0.59)	-3.40(-105, 3.69)	5.15(0.62, 9.67)	12.9(5.71, 20.2)	9.19(5.04, 13.3)	6.30(2.53, 10.1)	3.09 (1.12, 5.07)
Model 4	-4.93 (-11.1, 1.26)	-4.76 (-11.8, 2.28)	1.01 (-4.13, 5.93)	11.0 (3.89, 18.2)	6.83 (2.66, 10.9)	4.16 (0.09, 8.22)	3.98 (0.16, 4.07)

**SCAT (SD=5.9cm)**							
Crude model	25.1(10.4, 40.5)	19.7(0.26, 39.1)	4.85(-0.67, 10.4)	45.8(19.3, 72.4)	24.8(15.9, 33.7)	20.1(7.34, 32.9)	8.90 (4.59, 13.2)
Model 1	26.8(12.1, 41.6)	16.8(-2.42, 35.9)	3.83(-1.25, 8.90)	37.6(12.2, 63.0)	19.1(10.8, 27.5)	17.7(4.85, 30.5)	6.29 (2.34, 10.2)
Model 2	26.8 (12.1, 41.6)	18.2 (-0.79, 37.3)	3.64 (-1.41, 8.69)	35.9 (11.2, 60.6)	19.3 (10.6, 28.0)	17.7 (4.82, 30.6)	6.46 (2.47, 10.5)
Model 3	31.2(15.9, 46.6)	21.7 (1.92, 41.6)	10.6(3.76, 17.5)	12.4(-14.9, 39.6)	11.5(2.29, 20.6)	11.9(-1.71, 25.4)	9.78 (5.08, 14.5)
Model 4	14.8 (-3.80, 33.4)	10.9 (-10.2, 32.1)	7.66 (-0.45, 15.8)	0.54 (-26.6, 27.7)	9.70 (0.96, 18.4)	8.89 (-5.71, 23.5)	6.24 (1.14, 11.3)

Model 1: age, educational status and household SES; **Model 2:** Model 1 + alcohol, smoking and physical activity and **Model 3:** model 2 + other adiposity measures except those that were collinear. **Model 4:** Model 3 + glucose, HDL-C, LDL-C, SBP and HIV

Table S3: Output of meta-analysis pooling of main effect estimates of adiposity phenotype on CIMT using the fixed-effect inverse-variance model with heterogeneity measures

	**BMI in kg/m**	**WC in cm**	**HC in cm**	**VAT in cm**	**SCAT in cm**
Nanoro	18.8 (11.3, 26.5)	1.21 (0.70, 1.72)	1.19 (0.64, 1.75)	-4.12 (-7.82, -0.42)	13.0 (3.88, 22.2)
Navrongo	4.44 (-4.67, 13.55)	1.11 (0.48, 1.75)	0.28 (-0.32, 0.88)	2.59 (-2.09, 7.29)	7.40 (-3.88, 18.6)
Nairobi	12.1 (6.72, 17.6)	0.74 (0.39, 1.09)	0.69 (0.30, 1.09)	6.68 (4.03, 9.33)	3.63 (-3.19, 10.5)
Agincourt	18.5 (12.9, 23.9)	0.99 (0.67, 1.32)	1.22 (0.83, 1.62)	4.27 (1.99, 6.55)	0.27 (-2.47, 3.01)
Digkale	28.3 (21.1, 35.5)	1.71 (1.31, 2.11)	1.48 (1.05, 1.92)	10.2 (7.39, 12.9)	16.9 (10.3, 23.6)
Soweto	35.4 (28.2, 42.5)	2.15 (1.71, 2.58)	2.81 (2.23, 3.39)	10.8 (7.71, 13.9)	19.1 (11.9, 26.2)
Overall effect	19.5 (16.8, 22.3)	1.27 (1.09, 1.44)	1.23 (1.04, 1.42)	5.86 (4.65, 7.07)	5.00 (2.85, 7.15)
Test of overall effect	*z = 14.09, p<0.001*	*z = 14.78 p<0.001*	*z = 12.49 p<0.001*	*z = 9.512 p<0.001*	*z = 4.564 p<0.001*
Heterogeneity measues					
I^2	88.2%	84.4%	89.2%	90.2%	88.1%
Modified H^2	7.48	5.42	8.25	9.19	7.41
Tau^2	88.7	0.25	0.49	21.7	75.6

BMI, body mass index; WC, waist circumference; HC, hip circumference; VAT, visceral adipose tissue and SCAT, subcutaneous adipose tissue; I^2 = between-study variance (tau^2) as a percentage of total variance; Modified H^2 = ratio of tau^2 to typical within-study variance
